# Intervention in gene regulatory networks via greedy control policies based on long-run behavior

**DOI:** 10.1186/1752-0509-3-61

**Published:** 2009-06-15

**Authors:** Xiaoning Qian, Ivan Ivanov, Noushin Ghaffari, Edward R Dougherty

**Affiliations:** 1 Department of Electrical & Computer Engineering, Texas A&M University, College Station, TX, 77843, USA; 2 Department of Statistics, Texas A&M University, College Station, TX, 77843, USA; 3 Department of Veterinary Physiology & Pharmacology, Texas A&M University, College Station, TX, 77843, USA; 4 Computational Biology Division, Translational Genomics Research Institute, Phoenix, AZ, 85004, USA; 5 Department of Pathology, University of Texas M. D. Anderson Cancer Center, Houston, TX 77030, USA

## Abstract

**Background:**

A salient purpose for studying gene regulatory networks is to derive intervention strategies, the goals being to identify potential drug targets and design gene-based therapeutic intervention. Optimal stochastic control based on the transition probability matrix of the underlying Markov chain has been studied extensively for probabilistic Boolean networks. Optimization is based on minimization of a cost function and a key goal of control is to reduce the steady-state probability mass of undesirable network states. Owing to computational complexity, it is difficult to apply optimal control for large networks.

**Results:**

In this paper, we propose three new greedy stationary control policies by directly investigating the effects on the network long-run behavior. Similar to the recently proposed mean-first-passage-time (MFPT) control policy, these policies do not depend on minimization of a cost function and avoid the computational burden of dynamic programming. They can be used to design stationary control policies that avoid the need for a user-defined cost function because they are based directly on long-run network behavior; they can be used as an alternative to dynamic programming algorithms when the latter are computationally prohibitive; and they can be used to predict the best control gene with reduced computational complexity, even when one is employing dynamic programming to derive the final control policy. We compare the performance of these three greedy control policies and the MFPT policy using randomly generated probabilistic Boolean networks and give a preliminary example for intervening in a mammalian cell cycle network.

**Conclusion:**

The newly proposed control policies have better performance in general than the MFPT policy and, as indicated by the results on the mammalian cell cycle network, they can potentially serve as future gene therapeutic intervention strategies.

## Background

Boolean networks (BNs), and more generally, probabilistic Boolean networks (PBNs) [1,2], have been used for finding beneficial interventions in gene regulatory networks through the study of network dynamics. Upon describing these dynamics via Markov chains, optimal stochastic control policies can be determined via dynamic programming [3-5] to change the long-run dynamics, which are characterized by the steady-state distribution (SSD) of the network (Markov chain), the purpose being to reduce the risk of entering aberrant states and thereby alter the extant cell behavior. Three problems arise with this approach. First, the dynamic programming algorithm used to find optimal policies has complexity which increases exponentially with the number of the genes in the network. Approximate, or model-free, control policies have been proposed to alleviate this computational burden [6,7]. Second, the classical infinite-horizon approach to control requires a cost function and this requires subjective input. Third, and most importantly relative to the algorithms proposed in the current paper, optimization is with respect to the cost function and is only secondarily related to the steady-state distribution. Here, our purpose is to reduce the mass of the steady-state distribution corresponding to undesirable states and increase the mass corresponding to desirable states, and to do this directly without the mediating factor of a cost function.

In [7], a stationary mean-first-passage-time (MFPT) control policy is proposed that circumvents the need for a cost function and works directly with the transition probability of the Markov chain associated with the network. Biologically, it can be motivated by the following example. In a stable cancer cell line, the cells will keep proliferating without intervention. Assume that the goal of the intervention is to push the cell into programmed cell death (apoptosis) by intervening two candidate genes: p53 and telomerase. The p53 gene is the most well-known tumor suppressor gene [8-10]. The telomerase gene encodes telomerase, which maintains the integrity of the ends of chromosomes (telomeres) in germ cells and progenitor cells; therefore, it is responsible for replenishing cells during the normal cell turnover (homeostasis). In somatic cells, the telomerase gene is turned off, resulting in telomere shortening each time the cell divides – a key reason for the limited life-span of normal cells [11]. In the majority of tumor cells, telomerase is activated, which is believed to contribute to the prolonged life-span of the tumor cells [12]. This worsens prognosis for cancer patients [13,14]. Extensive experimental results indicate that when p53 is activated in the cells, for example in response to radiation, the cells undergo rapid growth inhibition and apoptosis in as short as a few hours [15]. In contrast, inhibition of the telomerase gene also leads to cell growth inhibition, differentiation, and cell death, but only after cells go through a number of cell divisions (allowing telomere shortening). Cell death takes a longer time through this latter process than through p53 activation. The use of mean first passage times for finding the best control gene is intuitive because the activation of p53 can lead more quickly (or with higher probability) to apoptosis than the inactivation of telemerase. Based on this kind of observation, the MFPT control policy employs two heuristics: (1) it is preferable to *reach *desirable states (apoptosis in the example) as early as possible; (2) it is preferable to *leave *undesirable states (cell proliferation) as early as possible.

Motivated by the success of the MFPT algorithm, in this paper, we propose three stationary control policies under the assumption that we have the transition probability matrix and the state transition diagram for the Markov chain of the network. Given that the intervention objective is to shift the steady-state distribution to desirable states, we propose to directly use the long-run behavior as our criterion for control instead of using the mean first passage time, which is an indirect measure. For the first control policy, we directly investigate the attractor states, which have been conjectured to correspond to phenotypes of the modeled cell. We replace the mean-first-passage-time criterion by the distance to (un)desirable attractor states of the underlying Markov chain, which can be computed efficiently. Since the shift of steady-state distribution can be computed efficiently using the analytic formula derived in [16-18], a second new control policy, having similar time complexity as the original MFPT control policy, is proposed based on the shift of steady-state distribution. A third policy also uses the steady-state distribution as the criterion, but gives up some computational efficiency in order to increase the certainty that applying the derived control policy will lead to the reduction of the total stationary mass for undesirable states. Because these new policies directly utilize the long-run characteristics of the network, we expect them to perform better than the MFPT policy with respect to shifting the steady-state distribution. A simulation study supports this expectation. In addition, a preliminary example of applying these control policies on a mammalian cell cycle network has shown that we can successfully identify gene E2F as the best potential control target, which has been conjectured in [19] through different mathematical modeling.

## Methods

### Background

#### Probabilistic Boolean networks

We focus on intervention in binary PBNs in this paper but these results directly extend to more general PBNs having any discrete range of values since the underlying models are always finite Markov chains. Following the standard definitions of the Boolean network model [1,2], PBNs are described by truth tables determined by Boolean regulatory rules and the related parameters, including various probabilities. In a binary Boolean network of *n *genes, the state of each gene *x*_*i *_∈ {0, 1} at time *t *+ 1 is determined by the values of a set *V*_*i *_of predictor genes at time *t *via a Boolean "predictor" function , where *K*_*i *_= |*V*_*i*_| denotes the number of predictor genes in *V*_*i *_and is called the input degree of *x*_*i *_in the network. Given a truth table, the network evolves as a trajectory of gene-expression vectors (states) **X**_*t *_∈ {0, 1}^*n*^, each known as a *gene activity profile *(GAP). From the initial state, a BN will eventually reach a set of states through which it will cycle forever. Each such set is called an *attractor cycle *and states within attractor cycles are *attractors*. The set of states leading to a specific attractor cycle is known as its *basin of attraction *(BOA).

For the stochastic extension of basic Boolean network model, we can have different variations. For Boolean networks with random perturbations (BNps), perturbation is introduced with a positive probability *p *by which the current state of each gene in the network can be randomly flipped. To further model the stochastic properties arising from either latent variables affecting network dynamics or the uncertainty from model inference, we allow *m *vector-valued network functions, **F **= {**f**_1_, **f**_2_,..., **f**_*m*_}, to determine the expression states of genes in the network model through time. We consider the network model which consists of a family {*B*_1_, *B*_2_,..., *B*_*m*_} of BNps governed by the corresponding functions, each BNp being referred to as a *context*. At any time point there is a positive probability *q *of switching the current governing context. Once a switch is called for at time point *t*, then one function from among **f**_1_,⋯, **f**_*m *_is randomly selected according to the probability distribution *c *= {*c*_1_,⋯, *c*_*m*_}, where it is possible for the current function to be chosen. There are two types of PBNs with different interpretations regarding *q*. If *q *< 1, the PBN is *context-sensitive *and *q *is usually assumed to be small. If *q *= 1, as in the original formulation of PBNs [2], the PBN is said to be *instantaneously random*. All of these various PBNs inherit the attractor cycles of their constituent BNs governed by predictor functions. Introduction of random perturbation makes the corresponding Markov chain of a PBN irreducible. Hence, it possesses a steady-state distribution *π *describing the long-run behavior. With sufficiently small *p*, *π *will reflect the attractor structure. For developing therapeutic intervention, we are especially interested in the proportion of time the network occupies an attractor in its steady state.

The dynamics of PBNs can be analyzed via their associated homogeneous irreducible finite Markov chains. For instantaneously random PBNs, the states of the associated Markov chain are the states (GAPs) of the network; for a context-sensitive PBN, the chain states are (context, GAP) pairs. We can derive the transition probability matrix *P *from the truth tables and the involved probabilistic parameters, and from there derive the steady-state distribution *π*. The computation of the transition probabilities between states in PBNs has been discussed in several papers [5,20,21]. We re-iterate them in the following theorem. The proof of the theorem can be referred to in Additional file 1.

**Theorem 1**: The transition probabilities from **y **to **x **for a BNp and an instantaneously random PBN are given by

(1)

where *η *(**x**, **y**) is the Hamming distance between **x **and **y**, and **1**_[**f**(**y**) = **x**] _is the indicator function that takes value 1 if **f**(**y**) = **x **according to the truth table and is equal to 0 otherwise; and

(2)

respectively. The transition probability from (*s*, **y**) to (*r*, **x**) for a context-sensitive PBN is given by

(3)

where *r*, *s *denote the *r*th and *s*th BNp, which are the BNps at time *t *+ 1 and *t*.

For a given transition matrix *P *for any type of PBN, we have

(4)

where *π *is the corresponding steady-state distribution for *P *and *T *denotes transpose.

In this paper, we focus on BNps and instantaneously random PBNs. Subsequently we will comment on extension to context-sensitive PBNs. For the moment, we note that, for the purposes of intervention, the reduction of a context-sensitive PBN to an instantaneously random PBN has been proposed in [5]. This reduction results in a large computational savings when deriving control strategies. Moreover, from the perspective of gene-expression inference of a context-sensitive PBN, the variables *r *and *s *in *P*_s, **y**_(*r*, **x**) are hidden variables because we only observe the transition of gene-expression states. Hence, it is difficult to accurately infer the transient structure of context-sensitive PBNs without a large amount of data [22]. In [21], the effect of the reduction from context-sensitive PBNs to instantaneously random PBNs in [5] is investigated and it is shown that, while there is some loss of control performance using the reduced model, the loss depending on the structure of the PBN, generally there can still be significant therapeutic benefits for these control strategies in situations where it is impractical to utilize the full model. Hence, our focus on the control policies on instantaneously random PBNs still leads to practical benefits to shift steady-state distributions beneficially.

#### Stochastic optimal intervention

The problem of optimal intervention for PBNs is formulated as an optimal stochastic control problem. Assuming that we can only control a single gene *g *in the network as in previous applications [5,7], the policy is of the form *u*_*g*_(*t*) ∈  = {0, 1}. If the control at time step *t *is on, *u*_*g*_(*t*) = 1, then the expression state for *g *is flipped; if *u*_*g*_(*t*) = 0, then the state of the control gene *g *remains unchanged. We consider the intervention as perturbing the transition probability of the original underlying Markov chain. Absent control, we have the transition probability *P*_***y***_(**x**) = *P *(**X**_*t*+1 _= **x**|**X**_*t *_= **y**); with control, we have , *u *∈ {0, 1}, where **X**_*t *_and *u*_*g*_(*t*) jointly determine . The new transition probability  decides the steady-state distribution of the underlying Markov chain for the controlled PBN. A natural way to intervene is to find a stationary control policy **u**_*g *_= {*u*_*g*_(**y**)|**y **∈ {0, 1}^*n*^} for all possible states **y **in the network so that the perturbed transition probabilities of the controlled Markov chain lead to the most beneficial steady-state distribution. In this way,

(5)

the policy being independent of time in this stationary policy. Because the size of the search space for this optimization problem is *O*(), the optimal solution  quickly becomes computationally infeasible as network size increases.

The previous algorithms [3-5] assign a cost function  for each intervention in the system and propose to solve the corresponding optimization problem in both finite and infinite horizon frameworks. In general, the cost depends on the state **y **at time *t*, the successor state **x **at time *t *+ 1, and the control input *u*. The expected immediate cost is defined for state **y**, when control *u *is selected, by



The *finite-horizon control *problem deals with control of the underlying Markov chain over a finite horizon and does not change its steady-state distribution. The *infinite-horizon control *problem finds the optimal stationary control policy  that is independent of time with respect to the expected total discounted cost and does affect the steady-state distribution. The discounting factor, *α *∈ (0, 1), ensures the convergence of the expected total cost over the long-run [23]. In the case of cancer therapy, the discounting factor emphasizes that obtaining treatment at an earlier stage is favored over later stages [7]. The expected total discounted cost formulation is given by

(6)

In this stochastic control problem, we seek an intervention strategy  among all the admissible intervention strategies Γ_*g *_that minimizes the above objective function for each state **X**_0 _= **y **in the network, i.e.,

(7)

Based on the results given in [23], it has been shown in [5] that an optimal intervention strategy exists for the discounted optimal stochastic control problem and the optimal cost function J* satisfies

(8)

J* is the unique solution of this equation within the class of bounded functions. Equation 8 is known as the *Bellman optimality equation*. An optimal control policy is a stationary policy that attains the minimum in the right-hand side of the Bellman optimality equation for all the states in the network.

From a strictly long-run perspective, absent any assigned costs, the preceding solution can be considered as an approximate way to find a control that most beneficially shifts the steady-state distribution. The problem here is that the algorithm requires appropriate settings for the cost function , which are difficult to obtain. Moreover, the existing iterative algorithms to find an optimal control policy in the above framework still have high computational complexity *O*(2^3*n*^) for each iteration [7].

#### Mean-first-passage-time (MFPT) control policy

In [7], a greedy stationary control policy using mean first passage times of the underlying Markov chain is proposed. When considering therapeutic interventions, the state space can be partitioned into the set *D *of desirable states and the set *U *of undesirable states according to the expression values of a given set of genes. Based on the intuition that an effective intervention strategy should reduce the likelihood of visiting undesirable states by increasing the time to reach undesirable states or decreasing the time to desirable states, a greedy mean-first-passage-time control policy can be derived.

To describe the MFPT control policy, without loss of generality, we assume that gene *x*_1 _decides **x **= {*x*_1_*x*_2_⋯*x*_*n*_} to be desirable when *x*_1 _= 1 and undesirable when *x*_1 _= 0. We also assume there is a set of control genes which are different from *x*_1_. For simplicity, and as is often done, we assume there is a single control gene denoted as *g*. The intuition behind the MFPT algorithm is that when a desirable **x **reaches *U *on average faster than **x**_*c*_, the state with the control gene *g *flipped from **x**, it is reasonable to apply control to flip *g *and start the next network transition from **x**_*c*_. The transition matrix of the original PBN can be written as



From general Markov chain theory [24], we can compute the mean first passage times *K*_*U *_and *K*_*D *_by solving the following system of linear equations:



where *e *denotes column vectors of 1's with the appropriate length; the vectors *K*_*U *_and *K*_*D *_contain the MFPTs from each state in *D *to *U*, and from each state in *U *to *D*, respectively.

To design the MFPT control policy, we check *K*_*D*_(**x**) - *K*_*D*_(**x**_*c*_) or *K*_*U *_(**x**_*c*_) - *K*_*U *_(**x**) for each state **x **and the corresponding flipped state **x**_*c*_. If **x **is undesirable, we check whether *K*_*D*_(**x**) - *K*_*D*_(**x**_*c*_) ≥ *λ *to make the time to reach the desirable states *D *faster; Otherwise when **x **is desirable, we check whether *K*_*U *_(**x**_*c*_) - *K*_*U *_(**x**) ≥ *λ *to make the time to leave the undesirable states *U *faster. The parameter *λ *is set to a higher value when the ratio of the cost of control to the cost of the undesirable states is higher, the intent being to apply the control less frequently; if we are not interested in limiting application of control, we set *λ *= 0. The computational complexity for finding the MFPT control policy in the original PBN is *O*(2^*n*^).

The pseudocode for the MFPT algorithm in Appendix 1, reproduced from [7], summarizes the procedure when applied to all possible control genes.

As noted in [7], even if one wishes to apply a cost-based optimization, the MFPT procedure can serve to find a good control gene and also gain insight on the controllability of the network.

### Control policies directly based on long-run behavior

#### Basin of Attraction (BOA) control policy

Although mean first passage time is closely related to the steady-state distribution, the MFPT control policy does not use the shift of stationary mass directly as a criterion. Given the basins of attraction (BOA), which determine the long-run behavior of a PBN, we can use this information to derive a control policy more directly related to the steady-state distribution. For this BOA control policy, we again assume that the state space is partitioned into the sets *D *and *U *of desirable and undesirable states. For any state **x**, let *A*^*j*^(**x**) be the set of attractors (the cycle) for the basin containing **x **in the *j*th constituent BNp, keeping in mind that a state **x **belongs to exactly one basin of attraction in each constituent BNp. Let , the union of attractors for the basins of **x **taken across all constituent BNps. Further, for each constituent BNp, we compute the minimal distance of each state to states in *D *or to states in *U*, and then we compute the respective PBN distances *d*_*D *_and *d*_*U *_by taking weighted averages with respect to the selection probabilities. Since most of the stationary mass is distributed in the attractors, the structural properties of the basins, including the properties of their attractors and their sizes, determine the long-run behavior and the steady-state distribution of the network [20]. Hence, it is reasonable to design a control policy based on the BOA structure.

We proceed in similar way to the manner in which the MFPT policy is derived in [7] to obtain a *basin of attraction (BOA) control policy*. For a pair of undesirable states **x **and **x**_*c*_, we first check whether *B*(**x**) or *B*(**x**_*c*_) contains any desirable attractors. If only one of them contains desirable attractors, then we decide the control policy to always go to that state so that we increase the likelihood of entering into desirable attractors. Otherwise, if both of them have desirable attractors or neither *B*(**x**) or *B*(**x**_*c*_) has desirable attractors, we compare *d*_*D*_(**x**) and *d*_*D*_(**x**_*c*_): Whichever is minimum, we apply control to get that state so as to reach the desirable states *D *faster. We do not apply any control if *d*_*D*_(**x**_*c*_) = *d*_*D*_(**x**). For a pair of desirable states **x **and **x**_*c*_, we first check whether *B*(**x**) or *B*(**x**_*c*_) contains any undesirable attractors. If only one of them contains undesirable attractors, then we apply control to flip to that state so that we reduce the risk of getting into undesirable attractors. If the condition is satisfied for both of the states or neither of them, we then check *d*_*U *_(**x**) and *d*_*U *_(**x**_*c*_) to make the time to reach the undesirable states *U *slower. The computational complexity for finding this control policy in the original PBN is *O*(2^*n*^), similar as in deriving the MFPT control policy. Since finding BOA structures of PBNs does not involve computing matrix inversions and is relatively less expensive than computing mean first passage times, especially with increasing number of genes in the network, the algorithm to find the BOA control policy is more efficient than the previous algorithm to find the MFPT control policy. The pseudocode in Appendix 2 summarizes the BOA procedure for finding the best control gene and the corresponding stationary control policy.

#### Steady-state distribution (SSD) control policy

Although the BOA structural properties constitute one determinant factor for the steady-state distribution that we aim to shift, the BOA algorithm does not use the steady-state distribution directly. Thus, we consider a control policy directly using the shifted stationary mass as the criterion of applying control. A key issue for such an algorithm is the efficient computation of the shifted stationary mass resulting from intervention. Recently, we have adapted the perturbation theory in finite Markov chains [18,25] to derive an analytic solution to compute the shifted mass efficiently [17]. We next state a theorem for general Markov chains which has appeared in [17,18,25].

**Theorem 2**: For a perturbed Markov chain with  = *P *+ *E *by a rank-one perturbation *E *= *ab*^*T*^, where *a *and *b *are two arbitrary vectors, the steady-state distribution is given by

(9)

where *e *is a vector with all its elements equal to 1, *t *and *u *are any vectors such that *π*^*T*^*t *≠ 0 and *u*^*T*^*e *≠ 0, and *β*^*T *^= *b*^*T *^[*I *- *P *+ *tu*^*T*^]^-1^.

If we let *t *= *e *and *u *= *π *in (9), we can derive

(10)

where *β*^*T *^= *b*^*T*^*Z *and *Z *is the fundamental matrix of the underlying Markov chain for the original network. Hence, the steady-state distribution of the rank-one perturbation is expressed in terms of *π *and *Z*, the steady-state distribution and fundamental matrix of the original network. Thus, for rank-one perturbations to regulatory functions, we have an explicit way to compute the exact shifted stationary mass.

Since the control by flipping one control gene *g *at any given state **x **simply changes the original transition matrix *P *to the controlled transition matrix  by replacing the row in *P *corresponding to the state **x **by the row that corresponds to the state **x**_*c *_with *g *flipped from **x**, the perturbation matrix can be written as a rank-one matrix and the perturbed steady-state distribution can be computed efficiently by:

(11)

where *p*_**x**_and  are the two rows corresponding to the states **x **and **x**_*c *_in *P*, *z*^**x**^is a column corresponding to the state **x **in *Z*, *π*_**x**_is the stationary mass for **x**, and (**x**) denotes the steady-state distribution after we apply gene flipping at the state **x**. Following this analytic solution, we can quickly compute the total stationary mass for undesirable states *π*_*U *_and  (**x**), and therefore the shifted mass after the possible controls to each state. Once we have that, we can derive a *steady-state distribution (SSD) control policy *based on a procedure similar to deriving the MFPT control policy. We compare the total stationary mass of undesirable states after applying control to **x **and **x**_*c*_: (**x**) and (**x**_*c*_). If both of them are larger than the original stationary mass, *π*_*U*_, of undesirable states, then we do not apply any control. Otherwise, we adopt the control on the state which leads to less stationary mass of the undesirable states. The computational complexity for finding this new control policy is again *O*(2^*n*^), while the complexity for each iteration in the algorithm increases from both the MFPT and BOA control policies a little bit by vector-matrix multiplications involved in (11). The pseudocode for the SSD algorithm in Appendix 3 summarizes the procedure for finding the best control gene and the corresponding stationary control policy based on shifted stationary mass.

As in the previous algorithms, deriving the SSD control policy only looks into the effects caused by perturbations to the pairs of states **x **and **x**_*c*_. Considering the perturbation to the original transition matrix *P*, for each network state **x**, we compare the steady-state distributions *π *and (**x**) that correspond to *P *and the controlled transition matrix  by replacing the row in *P *corresponding to the state **x **by the row that corresponds to the flipped state **x**_*c*_. If the undesirable stationary mass based on this one-row perturbation caused by flipping the control gene *g *at **x **reduces the undesirable stationary mass, then we decide the control policy for the state **x**: *u*_*g*_(**x**) = 1. For the derivation of the final stationary control policy for all the network states, we investigate the perturbation effects independently by studying the change from *P *to the corresponding controlled transition matrices by one-row perturbations at all the states: . The final control policy **u**_*g *_for the network actually leads to a multi-row perturbation to *P *by combining all the beneficial one row perturbations determined in the algorithm:. Generally,  is different from all the controlled transition matrices by one-row perturbations considered during the derivation of **u**_*g*_. Although, intuitively, this combination of beneficial one-row perturbations should reduce the total undesirable stationary mass, it is difficult to find the analytic characterization of the effect to the undesirable mass caused by the combination. We note here that in our simulations (**Results and Discussion**), the derived SSD control policy always reduces the stationary mass for undesirable states and performs better than the MFPT and BOA algorithms.

#### Conservative steady-state distribution (CSSD) control policy

All of the above control policies are relatively aggressive. Whenever we see a desirable difference by intervention through perturbing the original transition matrix of the network, we will apply control in a greedy manner. For single-gene control, the previous algorithms check whether applying gene flipping leads to immediate benefits. The decisions for different pairs of original and flipped states are independent. Therefore, we can derive the previous control policies for all the network states in a parallel fashion. However, there is no theoretical guarantee that applying these control policies will lead to the reduction of the stationary mass for undesirable states or, analogously, the increase of the stationary mass for desirable states. We now present a control policy, the *conservative steady-state distribution (CSSD) control policy*, for which we have a theoretical guarantee that the steady-state distribution after intervention will have less than or equal to the stationary mass of the undesirable states in the original network.

While we can compute the shifted steady-state distribution accurately using Theorem 2, we now introduce another theorem, proven in [17,25], which gives the new fundamental matrix  after applying a rank-one perturbation to the network.

**Theorem 3**: The fundamental matrix for the rank-one perturbed network  = *P *+ *E *= *P *+ *ab*^*T*^ is given by

(12)

Flipping one control gene at any given state **x**, similar to the derivation of (11), simply replaces the row in the original transition matrix corresponding to the state **x **by the row that corresponds to the flipped state **x**_*c*_. Hence, we have *a *= *e*_*x*_, which has 1 for the element corresponding to the state **x **and all 0's for the remaining elements in the vector; and . We can substitute these into (12) to compute the updated fundamental matrix.

Using this result, we now design a sequential algorithm that iteratively chooses states to control, so that we can theoretically guarantee that the control policy reduces the stationary mass of undesirable states. At each iteration, we check all the states, for which the control policies have not been decided, to see which state to control in order to achieve the largest reduction of the undesirable stationary mass. As in the previous subsection, for each state **x**, we compare the steady-state distributions *π *and (**x**) that correspond to *P *and the controlled transition matrix  by replacing the row in *P *corresponding to state **x **by the row that corresponds to the flipped state **x**_*c*_. Unlike deriving the SSD control policy *u*_*g*_(**x**) independently for all the states, for the CSSD control policy, we do not directly combine all the beneficial one-row perturbations into the new transition matrix  decided by the derived SSD control policy, In this new sequential CSSD algorithm, we check all possible one-row perturbations and find the best one row perturbation which results in the largest reduction of undesirable mass. Hence, we only select one state **x**^*best *^to control at each iteration if there is a reduction of undesirable mass. If we denote the controlled transition matrix by flipping the control gene *g *at that state as  at the *k*th iteration, the sequential algorithm in fact will generate a sequence of controlled transition matrices:

(13)

where *K *is the total number of iterations, and each pair of neighboring transition matrices differ by only one row. Here,  is the final controlled transition matrix with the derived control policy **u**_*g*_. As we obtain the controlled transition matrix at each iteration by a one-row perturbation to the previously computed controlled transition matrix, we can keep updating the exact steady-state distribution  and the fundamental matrix  using (11) and (12), respectively. Thus, at each iteration, we can directly compute the true stationary mass for undesirable states after intervention and make the decision about the control policy for the selected state as well. We let the algorithm run iteratively until we find that intervention to any state will actually increase the stationary mass of undesirable states from the previous iteration. In this way, we are guaranteed that the derived control policy will always have undesirable stationary mass less than or equal to that of the undesirable states in the original network. This algorithm is computationally more expensive compared with the previous algorithms as the search space is *O*(2^*n*^) at each iteration and the number of iterations *K *depends on the controllability of the networks. The advantage here is that the CSSD control policy is guaranteed to decrease undesirable stationary mass after intervention and, as well see in simulations, tends to outperform the SSD policy. The pseudocode in Appendix 4 summarizes the CSSD procedure for finding the best control gene and the corresponding stationary control policy.

The following theorem provides the guarantee that applying the conservative SSD algorithm [Appendix 4] will reduce the total undesirable stationary mass.

**Theorem 4**: The derived CSSD stationary control policy [Appendix 4] cannot increase the total undesirable stationary mass:

(14)

where *K *is the number of total iterations of the sequential algorithm.

**Proof**: We prove the theorem by induction. Starting with the first iteration *k *= 1, we always have  since we only apply the control when  as shown in Appendix 4. Now at the *k*th iteration, assuming , we want to show that . Indeed, in the CSSD algorithm [Appendix 4], at each iteration we apply the control only when . Hence, we have . *QED*

#### Extension to context-sensitive PBNs

We have restricted ourselves to BNps and instantaneously random PBNs till now. All the algorithms, including the MFPT algorithm, focus on the GAP space and this only corresponds to the Markov chain space for BNps and instantaneously random PBNs. However, these algorithms, including the MFPT algorithm, can be extended to intervene in context-sensitive PBNs with no theoretical obstacles. But as the state space changes from the space of GAPs to the space of (context, GAP) pairs in context-sensitive PBNs, the computational complexity of these algorithms will increase. Moreover, for the algorithms directly based on steady-state distributions, we have to apply iterative update schemes to compute the shifted steady-state distributions since the perturbations to the transition matrix become multiple-row perturbations [17].

## Results and discussion

### Comparison of four greedy control policies

In this section, as with the ensemble analysis in [7,26-28], we study the performance of the four greedy control policies, MFPT, BOA, SSD, and CSSD, based on a large number of randomly generated networks with similar network properties. The two most important parameters for generating random Boolean networks are the bias (*p*_*b*_) and connectivity (*K*). Here, *p*_*b *_is the mean of the *Bernoulli *distribution to generate the truth table of one Boolean function in a Boolean network, the bias *p*_*b *_being the probability that a randomly generated Boolean function takes on the value 1. *K *is the maximum input degree of the Boolean functions in the network. All simulation results in this section are based on 1, 000 randomly generated PBNs of 10 genes, including BNps and instantaneously random PBNs, with fixed *K *= 3 and different *p*_*b*_'s.

#### Performance comparison for BNps

**Figure 1:**
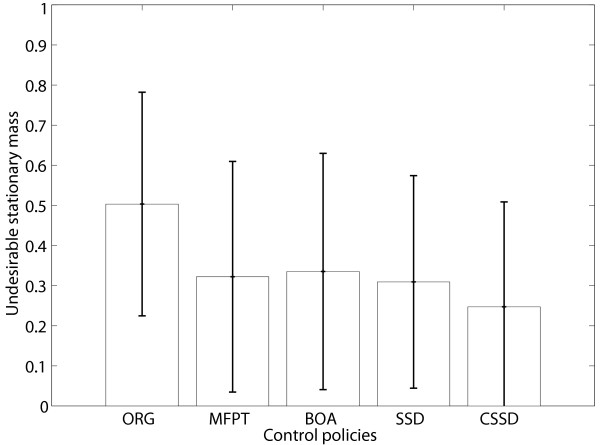
**Performance comparison for randomly generated BNps**. Performance comparison for 1000 randomly generated BNps with *p*_*b *_= 0.5 with respect to the means and standard deviations of the stationary masses for undesirable states for different control policies: ORG – original undesirable stationary mass; MFPT – undesirable stationary mass after applying the MFPT control policy; BOA – undesirable stationary mass after applying the BOA control policy; SSD – undesirable stationary mass after applying the steady-state distribution control policy; CSSD – undesirable stationary mass after applying the conservative SSD control policy.

We first consider performance comparison using randomly generated BNps with 10 genes and *p *= 0.01 in all experiments. Each BN is randomly generated with a specific bias *p*_*b*_. Since the bias affects the dynamical properties of randomly generated BNs [27], it is taken as a parameter in our simulations. The bias *p*_*b *_of each BNp is randomly selected from a *beta *distribution. The mean of the beta distribution varies 0.1 to 0.9 with step-size 0.2. The variance of the beta distribution is 0.000064. We generate 1000 random BNps for each bias mean. For each network, without loss of generality, undesirable and desirable states are defined by *x*_1 _= 0 and *x*_1 _= 1, respectively, and the control gene is *x*_10_. All four control policies are applied: MFPT with *λ *= 0; BOA; SSD; and CSSD. Table 1 summarizes the average stationary mass for the undesirable states before control (ORG) and after applying these four different policies. Note that the undesirable stationary mass before control is dependent on *p*_*b*_. Figure 1 shows both the means and standard deviations of the stationary mass for undesirable states, , with *p*_*b *_= 0.5 before and after control. From both the table and the figure, we see that the CSSD control policy has the best performance and the SSD policy also achieves better performance compared with the MFPT and BOA policies. They also show that in average, the BOA policy performs similarly to the MFPT policy.

**Table 1:** Performance comparison for randomly generated BNps.

Control policies	*p*_*b*_				
	
	0.1	0.3	0.5	0.7	0.9
ORG	0.8923	0.7000	0.5034	0.2781	0.1110
MFPT	0.8641	0.5572	0.3222	0.1574	0.0763
BOA	0.8644	0.5717	0.3352	0.1657	0.0777
SSD	0.8609	0.5415	0.3093	0.1491	0.0748
CSSD	0.8594	0.5102	0.2472	0.1308	0.0743

Average  over 1000 randomly generated BNps before and after applying all of four control policies: ORG – original stationary mass for undesirable states before control; MFPT – mean-first-passage-time control policy; BOA – BOA control policy; SSD – steady-state distribution control policy; CSSD – conservative SSD control policy with x_10_ as the control gene.

Recall that it is guaranteed that the CSSD control policy always leads to a reduction of the stationary mass for undesirable states. Table 2 gives the percentages of random BNps with stationary mass shift Δ = *π*_*U *_-  ≥ 0 for different control policies and different values of *p*_*b*_. The MFPT control policy is probably the most aggressive policy with *λ *= 0 because the algorithm will force gene flipping whenever a difference between the mean first passage times is observed, and this aggressiveness is reflected by the lowest percentage in Table 2. As must be the case, the CSSD policy always leads to a reduction of the undesirable stationary mass. In this simulation, the SSD policy also always reduces the undesirable mass, although we do not have a proof that this is always the case. In fact, we have tried different settings with *K *and *p*_*b *_and for all tested settings, the SSD control policy never increases the undesirable mass. It would be nice to find a mathematical way to prove that the SSD control policy has the guarantee that it will always shift the stationary mass beneficially. In general, the BOA, SSD, and CSSD control policies are all relatively conservative compared to the MFPT policy since the criteria they use are directly related to the network's long-run behavior. It is also interesting to see that there is some correlation of the percentages with *p*_*b*_.

**Table 2:** Percentages of random BNps with improved performance after applying control.

Control policies	*p*_*b*_				
	
	0.1	0.3	0.5	0.7	0.9
MFPT	96.4%	91.1%	90.6%	91.1%	97.6%
BOA	100.0%	99.4%	98.4%	99.5%	100.0%
SSD	100.0%	100.0%	100.0%	100.0%	100.0%
CSSD	100.0%	100.0%	100.0%	100.0%	100.0%

Percentages of random BNps with Δ = π*_U _*-  ≥ 0 within 1000 random BNps after applying four control policies with x_10_ as the control gene.

#### Performance comparison for instantaneously random PBNs

**Figure 2:**
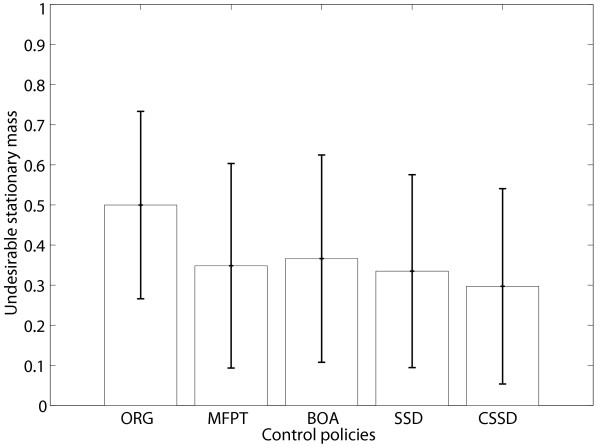
**Performance comparison for randomly generated instantaneously random PBNs**. Performance comparison for 1000 randomly generated instantaneously random PBNs with *p*_*b *_= 0.5 with respect to the means and standard deviations of the stationary masses for undesirable states for different control policies: ORG – original undesirable stationary mass; MFPT – undesirable stationary mass after applying the MFPT control policy; BOA – undesirable stationary mass after applying the BOA control policy; SSD – undesirable stationary mass after applying the steady-state distribution control policy; CSSD – undesirable stationary mass after applying the conservative SSD control policy.

We have also compared the performances using 1000 randomly generated instantaneously random 10-gene PBNs with 2 context BNps, generated similarly as the BNps in the previous subsection. We fix the perturbation probability at *p *= 0.01. The selection probabilities are *c*_1 _= *c*_2 _= 0.5. We again define states with *x*_1 _= 0 as undesirable and states with *x*_1 _= 1 as desirable, and again apply all four control policies with *x*_10 _as the control gene. Table 3 summarizes the average stationary mass for the undesirable states before control (ORG) and after applying these four different control policies. The means and standard deviations of the stationary mass for undesirable states  with *p*_*b *_= 0.5 before and after control are shown in Fig. 2. The CSSD policy has the best performance and the SSD policy also achieves better performance compared with the MFPT and BOA policies, as in the simulations for BNps.

**Table 3:** Performance comparison for randomly generated instantaneously random PBNs.

Control policies	*p*_*b*_				
	
	0.1	0.3	0.5	0.7	0.9
ORG	0.8939	0.6934	0.4997	0.2967	0.1063
MFPT	0.8567	0.5807	0.3484	0.1912	0.0747
BOA	0.8595	0.5966	0.3662	0.2026	0.0784
SSD	0.8547	0.5637	0.3349	0.1822	0.0728
CSSD	0.8525	0.5439	0.2971	0.1670	0.0723

Average  over 1000 randomly generated PBNs before and after applying all of four control policies: ORG – original stationary mass for undesirable states before control; MFPT – mean-first-passage-time control policy; BOA – BOA control policy; SSD – steady-state distribution control policy; CSSD – conservative SSD control policy with x_10_ as the control gene.

We show the percentages of random PBNs with stationary mass shift Δ = *π*_*U *_-  ≥ 0 in Table 4 for different control policies and different *p*_*b*_'s. We see a similar trend as with the simulations for BNps.

**Table 4:** Percentages of random PBNs with improved performance after applying control.

Control policies	*p*_*b*_				
	
	0.1	0.3	0.5	0.7	0.9
MFPT	94.4%	91.3%	94.4%	92.5%	95.6%
BOA	99.5%	96.1%	97.0%	94.9%	99.6%
SSD	100.0%	100.0%	100.0%	100.0%	100.0%
CSSD	100.0%	100.0%	100.0%	100.0%	100.0%

Percentages of random PBNs with Δ = π*_U _*-  ≥ 0 within 1000 random PBNs after applying four control policies with x_10_ as the control gene.

We observe similar trends for randomly generated networks with different numbers of genes [see Additional file 1]. Finally, while the MFPT and BOA control policies are close in terms of the shift of undesirable stationary mass, the MFPT control policy being slightly better in many cases, the BOA control policy is always significantly better than the MFPT control policy in terms of producing a beneficial shift.

### A mammalian cell cycle network

We now apply these different control policies on a probabilistic Boolean network (PBN) model of the mammalian cell cycle recently proposed in [29]. For a normal mammalian organism, cell division coordinates with overall growth controlled via extra-cellular signals. These signals indicate whether a cell should divide or remain in a resting state. The positive signals, or growth factors, instigate the activation of Cyclin D (CycD), which is one of the key genes in the mammalian cell cycle. The other two important genes are retinoblastoma (Rb) and p27. Rb is a tumor-suppressor gene. This gene is expressed in the absence of the cyclins, which inhibit Rb by phosphorylation. Gene p27 is also active in the absence of the cyclins. Whenever p27 is present, it blocks the action of CycE or CycA and Rb can also be expressed, even in the presence of CycE or CycA. Hence, it stops the cell cycle.

The preceding explanation represents the wild-type cell cycle model. In this model, when p27 is active, the cell cycle can be stopped in cancerous situations. When we follow one of the proposed mutations in [29], in which p27 is mutated and it is always off, the mutation introduces a situation where both CycD and Rb might be inactive. As a result, in this mutated phenotype, the cell cycles in the absence of any growth factor. In other words, we consider the logical states in which both Rb and CycD are down-regulated as undesirable states.

**Figure 3:**
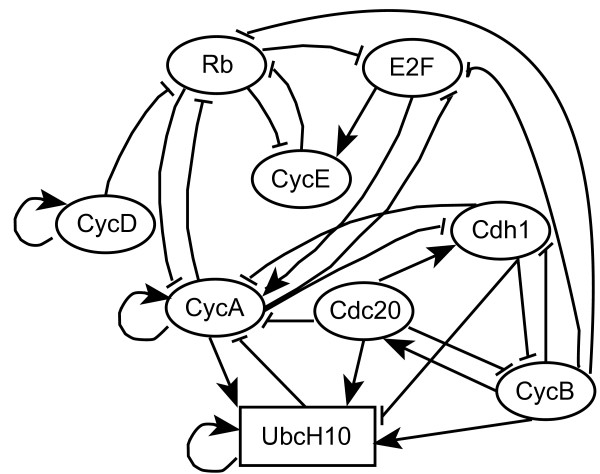
**Mutated mammalian cell cycle network**. Logical regulatory graph for the mutated mammalian cell cycle network (modified from Fig. 1 in [29]). Blunt arrows stand for inhibitory effects; normal arrows for activations.

We use the PBN that postulates the cell cycles with mutated phenotype in our experiments. We construct the instantaneously random PBN of the cell cycle based on the Boolean functions in Table 5 with mutated p27. This PBN consists of 9 genes: CycD, Rb, E2F, CycE, CycA, Cdc20, Cdh1, UbcH10, and CycB. The illustration of the relationship between these genes in the PBN is shown in Fig. 3. The above order of genes is used in the binary representation of the logical states, with CycD as the most significant bit and CycB as the least significant bit. The order of genes in the logical states does not affect our analysis or intervention. We assume that the extra-cellular signal to the cell cycle model is a latent variable. The growth factor is not part of the cell and its value is determined by the surrounding cells. The expression of CycD changes independently of the cell's content and reflects the state of the growth factor. Depending on the expression status of CycD, we obtain two constituent Boolean networks. The first constituent Boolean network is determined based on the Boolean functions in Table 5 when the value of CycD is equal to 0. Similarly, the second constituent Boolean network is determined by setting the value of CycD to 1. To completely define the PBN, we set the perturbation probability *p *= 0.01, and the probability of selecting each constituent Boolean network *c*_*j *_= 0.5, *j *= 1, 2.

**Table 5:** Definitions of Boolean functions for the mutated mammalian cell cycle PBN.

Order	Gene	Regulating function
*x*_1_	*CycD*	extra-cellular signals
*x*_2_	*Rb*	
*x*_3_	*E*2*F*	
*x*_4_	*CycE*	
*x*_5_	*CycA*	
*x*_6_	*Cdc*20	*CycB*
*x*_7_	*Cdh*1	
*x*_8_	*UbcH*10	**
*x*_9_	*CycB*	

Definitions of Boolean functions for the mutated 9-gene mammalian cell cycle PBN.

**Figure 4:**
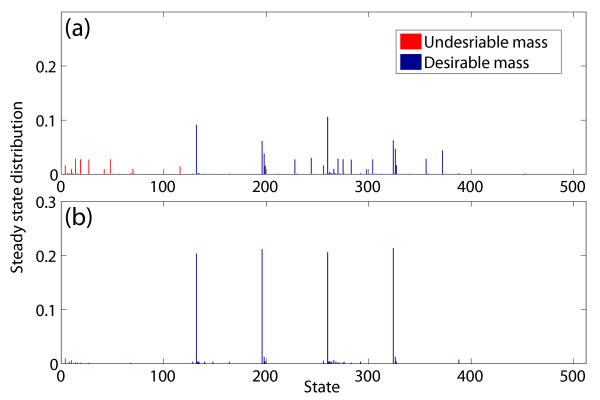
**Steady-state distribution shifts for the mutated mammalian cell cycle PBN**. Steady-state distribution shifts for the mutated 9-gene mammalian cell cycle PBN with *p *= 0.01: (a) Original steady-state distribution; (b) Steady-state distribution after applying the conservative SSD control policy with E2F as the control gene.

We first compute the steady-state distribution for this mutated PBN as shown in Fig. 4(a). Since the logical states in which both Rb and CycD are down-regulated are undesirable states, we compute the total stationary mass for the undesirable states: . We then apply the MFPT control policy with *λ *= 0.1, the BOA control policy, the SSD control policy, and CSSD control policy to find the single control gene to reduce the stationary mass for the undesirable states. As CycD and Rb are two genes deciding network states to be either desirable or undesirable, it is problematic to apply the MFPT control policy if these genes are considered. Hence, in the experiments, we only check the last 7 genes for all four control policies. Table 6 gives the total stationary masses for different control genes using these control policies. From the table, all the control policies find the same best control gene, E2F, and their performances are similar. However, when we compare the total stationary masses of the undesirable states for all possible control genes, we see that the performance of the CSSD control policy is the best among all the control policies. The SSD control policy also gives superior performance. Figure 4(b) shows the steady-state distribution after applying the derived CSSD control policy for the best control gene E2F.

**Table 6:** Total stationary mass of undesirable states after applying control policies for all the potential target genes in the mutated mammalian cell cycle PBN.

Control policies	Potential control genes						
	
	E2F	CycE	CycA	Cdc20	Cdh1	UbcH10	CycB
MFPT	0.0445	0.1534	0.1799	0.2003	0.1399	0.2037	0.1320
BOA	0.0505	0.1534	0.2092	0.1832	0.1912	0.2161	0.1712
SSD	0.0386	0.1534	0.1784	0.1614	0.1369	0.2025	0.1531
CSSD	0.0386	0.1534	0.1770	0.1371	0.1369	0.2025	0.1303

after applying all of four control policies for all the potential control genes in the mutated 9-gene PBN.

A recent paper suggests that the Myc-Rb-E2F pathway functions as a bistable switch that separates quiescence and proliferation for the mammalian cell cycle [19]. The paper shows that E2F activation correlates directly with the ability of a cell to reverse the R(estriction)-point, which marks the critical event when a mammalian cell commits to proliferation independent of growth stimulation. The R-point is fundamental for normal differentiation and appears to be dysregulated in virtually all cancers. It is interesting to see that through different mathematical modeling we reach the same conclusion that E2F is the best potential target for future gene therapy design.

We have also computed the time to find the best control gene for all of four control policies. The values in Table 7 follow roughly the time complexity predicted in **Methods **section. Note that we collected the running time with the unoptimized code running in MATLAB on a standard PC with a 1.8*GHz *CPU and 1*Gb *memory. These values only serve as rough indices to show that the first three greedy control policies have roughly the same time complexity.

**Table 7:** Running time for deriving four control policies in the mutated mammalian cell cycle PBN.

Control policies	MFPT	BOA	SSD	CSSD
Running time (sec.)	27.9033	27.8034	28.0793	599.7273

Running time (measured in seconds) for deriving four control policies for all the potential control genes in the mutated 9-gene PBN.

## Conclusion

In this paper, we propose three new greedy stationary control policies directly using long-run behavior change by intervention to define the control criteria. Through simulations, we have shown that the MFPT, BOA, and SSD policies perform similarly with respect to computational complexity and all reduce the risk of entering undesirable states that correspond to aberrant phenotypes of the modeled cells, with the SSD policy having better average performance in this regard than the other two. For the conservative CSSD policy, we are guaranteed that intervention will lead to beneficial shift of steady-state distributions. We have also illustrated how these control policies can serve as the potential gene therapeutic intervention strategies in the future with a mammalian cell cycle network. As with the MFPT control policy, not only can they be used to directly shift the steady-state distribution without the need for a cost function, they can also be used to predict the potential control gene of the network, serve as reduced-complexity approximations to cost-based control policies, and provide measures of network controllability. Our future direction will be focused on understanding the performance of these greedy control policies relative to their robustness in the presence of inaccurate inference [30] and network reduction [31,32].

## Authors' contributions

XQ conceived the study, developed the algorithms, performed the simulations, and wrote the manuscript. II collaborated in the analysis, and helped draft the manuscript. NG helped draft the manuscript. ERD conceived the study, participated in the analysis and interpretation of the results, and helped draft the manuscript. All authors read and approved the final manuscript.

## Appendix

### Appendix 1 – MFPT algorithm [7]

Partition the state-space into undesirable *U *and desirable *D *subsets.

Compute *K*_*U *_and *K*_*D*_.

*g *← 1.

repeat

   **for **All states **x **in *U ***do**

      **x**_*c *_← flip control gene *g *in **x**.

      **if ***K*_*D*_(**x**) - *K*_*D*_(**x**_*c*_) > *λ ***then**

         *u*_*g*_(**x**) = 1;

      **else**

         *u*_*g*_(**x**) = 0;

      **end if**

   **end for**

   **for **All states **x **in *D ***do**

      **x**_*c *_← flip control gene *g *in **x**.

      **if ***K*_*U *_(**x**_*c*_) - *K*_*U *_(**x**) > *λ ***then**

         *u*_*g*_(**x**) = 1;

      **else**

         *u*_*g*_(**x**) = 0;

      **end if**

   **end for**

   *g *← *g *+ 1.

**until ***g *> number of genes

### Appendix 2 – BOA algorithm

Partition the state-space into undesirable *U *and desirable *D *subsets.

Determine the BOA structure of the network, including *B*(**x**), *d*_*D*_(**x**) or *d*_*U*_(**x**) for each state **x**. *g *← 1.

repeat

   **for **All states **x **in *U ***do**

      **x**_*c *_← flip control gene *g *in **x**.

      **if ***B*(**x**) contains no desirable attractors &&*B*(**x**_*c*_) contains desirable attractors **then**

         *u*_*g*_(**x**) = 1;

      **else**

         **if ***d*_*D*_(**x**) > *d*_*D*_(**x**_*c*_) **then**

            *u*_*g*_(**x**) = 1;

         **else**

            *u*_*g*_(**x**) = 0;

         **end if**

      **end if**

   **end for**

   **for **All states **x **in *D ***do**

      **x**_*c *_← flip control gene *g *in **x**.

      **if ***B*(**x**) contains undesirable attractors &&*B*(**x**_*c*_) contains no undesirable attractors **then**

         *u*_*g*_(**x**) = 1;

      **else**

         **if ***d*_*U *_(**x**_*c*_) > *d*_*U *_(**x**) **then**

            *u*_*g*_(**x**) = 1;

         **else**

            *u*_*g*_(**x**) = 0;

         **end if**

      **end if**

   **end for**

   *g *← *g *+ 1.

**until ***g *> number of genes

### Appendix 3 – SSD algorithm

Partition the state-space into undesirable *U *and desirable *D *subsets.

Compute the original steady-state distribution *π *and the fundamental matrix *Z*.

*g *← 1.

repeat

   **for **All pairs of states **x **and **x**_*c *_← flip control gene *g *in **x do**

      Compute  and  using (11).

      **if ****then**

         *u*_*g*_(**x**) = 0;

         *u*_*g*_(**x**_*c*_) = 0;

      **else**

         **if ****then**

            *u*_*g*_(**x**) = 1;

            *u*_*g*_(**x**_*c*_) = 0;

         **else**

            *u*_*g*_(**x**) = 0;

            *u*_*g*_(**x**_*c*_) = 1;

         **end if**

      **end if**

   **end for**

   Compute the shifted stationary mass of undesirable states , where  is the stationary mass of the undesirable states after we apply the derived control policy;

   *g *← *g *+ 1.

**until ***g *> number of genes

### Appendix 4 – Conservative SSD algorithm

Partition the state-space into undesirable *U *and desirable *D *subsets.

Compute the original steady-state distribution *π *and the fundamental matrix *Z*.

*g *← 1.

repeat

   *π*^*best *^= *p*;

   *Z*^*best *^= *Z*;

   Assign the set of states with no control assigned as *L*;

   **repeat**

      Δ^*best *^= 0;

      **for **All states **x **∈ *L ***do**

         Compute  with *π*^*best *^and *Z*^*best *^based on (11);

         **if **Δ*π*_*U *_> Δ^*best *^**then**

            Δ^*best *^= Δ*π*_*U*_;

            Assign the best state to control: **x**^*best *^= **x**;

            *π*^*best *^= ;

         **end if**

      **end for**

      **if **Δ^*best *^> 0 **then**

         *u*_*g*_(**x**^*best*^) = 1;

         *u*_*g*_() = 0;

         *L *= *L*\{**x**^*best*^, };

         Compute *Z*^*best *^based on (12) accordingly;

      **end if**

   **until **Δ^*best *^= 0

   Δ(*g*) = *π*_*U *_^- ^;

   *g *← *g *+ 1.

**until ***g *> number of genes

## Supplementary Material

Additional file 1 **"supplement.pdf" – Supplementary file for "Intervention in gene regulatory networks via greedy control policies based on long-run behavior"**. The file "supplement.pdf" contains the proof for Theorem 1 and the additional simulation results for 1000 randomly generated BNps and instantaneously random PBNs with different number of genes and different perturbation probability *p*. The performance comparison for four stationary control policies – mean-first-passage-time (MFPT) control policy; BOA control policy; steady-state distribution (SSD) control policy; and conservative steady-state distribution (CSSD) control policy – leads to the same conclusions that we have discussed in the manuscript. Click here for file
