# Supplementary material for: Intervention in gene regulatory networks via greedy control policies based on long-run behavior
Source: BMC Syst Biol. 2009 Jun 15;3:61. doi: 10.1186/1752-0509-3-61 (PMC2728102; doi:10.1186/1752-0509-3-61)
Supplement: Additional file 1 — "supplement.pdf" – Supplementary file for "Intervention in gene regulatory networks via greedy control policies based on long-run behavior". The file "supplement.pdf" contains the proof for Theorem 1 and the additional simulation results for 1000 randomly generated BNps and instantaneously random PBNs with different number of genes and different perturbation probability p. The performance comparison for four stationary control policies – mean-first-passage-time (MFPT) control policy; BOA control policy; steady-state distribution (SSD) control policy; and conservative steady-state distribution (CSSD) control policy – leads to the same conclusions that we have discussed in the manuscript. [file 1752-0509-3-61-S1.pdf]

This supplementary file provides the supplementary materials for the manuscript **"Intervention in gene regulatory networks via greedy control policies based on long-run behavior"**, including the relevant theorem and the additional simulation results.

*** Note:** The order of the references in this file is *not* identical to the order in the manuscript. The list of references cited in this file can be found on the last page.

Theorems and Proofs

Theorem 1

The computation of the transition probabilities between states in PBNs has been discussed in several papers [Brun *et al.*, 2005, Faryabi *et al.*, 2009, Pal *et al.*, 2006]. We re-iterate them in the following theorem:

Theorem 1: The transition probabilities from $\mathbf{y}$ to $\mathbf{x}$ for a BNp and an instantaneously random PBN are given by

$$P_{\mathbf{y}}(\mathbf{x}) = \mathbf{1}_{[\mathbf{f}(\mathbf{y})=\mathbf{x}]}(1-p)^n + \mathbf{1}_{[\mathbf{x}\neq\mathbf{y}]}p^{\eta(\mathbf{x},\mathbf{y})}(1-p)^{n-\eta(\mathbf{x},\mathbf{y})}, \tag{1}$$

where $\eta(\mathbf{x},\mathbf{y})$ is the Hamming distance between $\mathbf{x}$ and $\mathbf{y}$, and $\mathbf{1}_{[\mathbf{f}(\mathbf{y})=\mathbf{x}]}$ is the indicator function that takes value 1 if $\mathbf{f}(\mathbf{y}) = \mathbf{x}$ according to the truth table and is equal to 0 otherwise; and

$$P_{\mathbf{y}}(\mathbf{x}) = \sum_{j=1}^{m} c_j \mathbf{1}_{[\mathbf{f}_j(\mathbf{y})=\mathbf{x}]}(1-p)^n + \mathbf{1}_{[\mathbf{x}\neq\mathbf{y}]}p^{\eta(\mathbf{x},\mathbf{y})}(1-p)^{n-\eta(\mathbf{x},\mathbf{y})}, \tag{2}$$

respectively. The transition probability from $(s,\mathbf{y})$ to $(r,\mathbf{x})$ for a context-sensitive PBN is given by

$$\begin{aligned} P_{s,\mathbf{y}}(r,\mathbf{x}) &= \mathbf{1}_{[r=s]}((1-q)+qc_s)\{\mathbf{1}_{[\mathbf{f}_s(\mathbf{y})=\mathbf{x}]}(1-p)^n + \mathbf{1}_{[\mathbf{x}\neq\mathbf{y}]}p^{\eta(\mathbf{x},\mathbf{y})}(1-p)^{n-\eta(\mathbf{x},\mathbf{y})}\} \\ &+ \mathbf{1}_{[r\neq s]}qc_r\{\mathbf{1}_{[\mathbf{f}_r(\mathbf{y})=\mathbf{x}]}(1-p)^n + \mathbf{1}_{[\mathbf{x}\neq\mathbf{y}]}p^{\eta(\mathbf{x},\mathbf{y})}(1-p)^{n-\eta(\mathbf{x},\mathbf{y})}\}, \end{aligned} \tag{3}$$

where r, s denote the rth and sth BNp, which are the BNps at time $t+1$ and t.

Proof: With perturbation, the dynamics of a BNp can be analyzed as a homogeneous irreducible finite Markov chain $\mathbf{X}_t$ with the state space $\{0,1\}^n$. The state transitions of a BNp over time are governed by transition rules $\mathbf{f}$ and perturbation. Implicitly, we assume that there is an i.i.d. random perturbation vector $\gamma \in \{0,1\}^n$, where the ith gene flips if the ith component of γ is equal to 1. Here, $p = P\{\gamma_i = 1\} = E[\gamma_i]$ is the same for all the genes. If $\mathbf{X}_t \in \{0,1\}^n$ is the state of the network at time t, then the next state $\mathbf{X}_{t+1}$ is either $\mathbf{f}(\mathbf{X}_t)$ with probability of $(1-p)^n$ or $\mathbf{X}_t \oplus \gamma$ with probability $1-(1-p)^n$ (at least one perturbation), where $\oplus$ is component-wise addition modulo 2. For each pair of states $\mathbf{x}$ and $\mathbf{y}$, We write the transition probability from $\mathbf{y}$ to $\mathbf{x}$ at arbitrary time t as $P_{\mathbf{y}}(\mathbf{x}) = P[\mathbf{X}_{t+1} = \mathbf{x}|\mathbf{X}_t = \mathbf{y}]$. This probability is a weighted sum of two transition probabilities:

$$P_{\mathbf{y}}(\mathbf{x}) = \mathbf{1}_{[\mathbf{f}(\mathbf{y})=\mathbf{x}]}(1-p)^n + p_{\mathbf{y}}(\mathbf{x})[1-(1-p)^n], \tag{4}$$

where $\mathbf{1}_{[\mathbf{f}(\mathbf{y})=\mathbf{x}]}$ is the indicator function that takes value 1 if $\mathbf{f}(\mathbf{y}) = \mathbf{x}$ according to the truth table and is equal to 0 otherwise, and $p_{\mathbf{y}}(\mathbf{x})$ is the probability that the perturbation moves from $\mathbf{y}$ to $\mathbf{x}$. With $\eta(\mathbf{x}, \mathbf{y})$ as the Hamming distance between $\mathbf{x}$ and $\mathbf{y}$, we can write:

$$p_{\mathbf{y}}(\mathbf{x}) = \mathbf{1}_{[\mathbf{x} \neq \mathbf{y}]} \frac{p^{\eta(\mathbf{x},\mathbf{y})}(1-p)^{n-\eta(\mathbf{x},\mathbf{y})}}{1-(1-p)^n}.$$

Plugging this into (4), we obtain (1). Based on this, we can derive the transition matrix P of the underlying Markov chain by computing all of its matrix entries.

For instantaneously random PBNs, we assume that we select one function from among $\mathbf{f}_1, \cdots, \mathbf{f}_m$, representing one of constituent BNps, with probabilities $\mathbf{c} = \{c_1, \cdots, c_m\}$ at any time point t. Therefore, the transition probability for the state pair $\mathbf{x}$ and $\mathbf{y}$ can be written according to (2).

The transition probability for context-sensitive PBNs is more complicated because $P_{\mathbf{y}}(\mathbf{x})$ depends on the function $\mathbf{f}_j$ selected at time t. The state space of the underlying Markov chain for context-sensitive PBNs is in fact the joint space of the gene expression states and the constituent BNps. Thus, the transition probability of the underlying homogeneous Markov chain $(B_t, \mathbf{X}_t)$ is given by (3), where r, s denote the rth and sth BNp, which are the BNps at time $t+1$ and t. ■

Comparison of four greedy control policies on random networks

This file contains the simulation results for randomly generated BNps and instantaneously random PBNs with 7 and 10 genes, where we change the perturbation probability p to check its effects on control performance. The random BNps and PBNs are generated similarly as the 10–gene randomly generated PBNs in the manuscript.

Performance comparison for BNps

7-gene random BNps

We consider performance comparison using randomly generated BNps with 7 genes and $p = 0.01$ first. For each network, without loss of generality, undesirable and desirable states are defined by $x_1 = 0$ and $x_1 = 1$, respectively, and the control gene is x_7. All four control policies are applied: MFPT with $\lambda = 0$; BOA; SSD; and CSSD. Table 1 summarizes the average stationary mass for the undesirable states before control (ORG) and after applying these four different policies. Figure. 1 shows both the means and standard deviations of the stationary mass for undesirable states $\sum_{x_1=0} \pi_{\mathbf{x}}$ with $p_b = 0.5$ before and after control. From both the table and the figure, we see that the CSSD control policy has the best performance and the SSD policy also achieves better performance compared with the MFPT and BOA policies. They also show that in average, the BOA policy performs similarly to the MFPT policy.

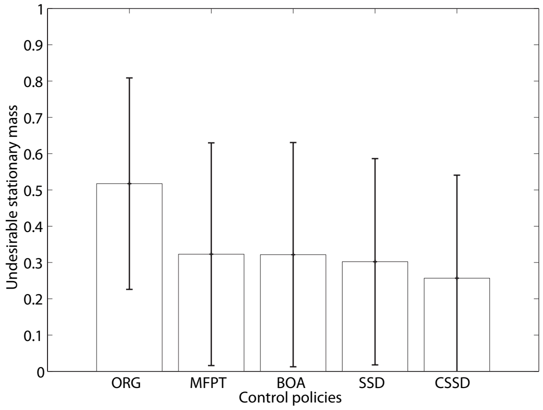

Figure 1: Performance comparison for 1000 randomly generated BNps with $p_b = 0.5$ with respect to the means and standard deviations of the stationary masses for undesirable states for different control policies: ORG – original undesirable stationary mass; MFPT – undesirable stationary mass after applying the MFPT control policy; BOA – undesirable stationary mass after applying the BOA control policy; SSD – undesirable stationary mass after applying the steady-state distribution control policy; CSSD – undesirable stationary mass after applying the conservative SSD control policy.

Table 2 gives the percentages of random BNps with stationary mass shift $\Delta = \pi_U - \tilde{\pi}_U \geq 0$ for different control policies and different values of p_b. The MFPT control policy is probably the most aggressive policy with $\lambda = 0$ because the algorithm will force gene flipping whenever a difference between the mean first passage times is observed, and this aggressiveness is reflected by the lowest percentage in Table 2. As must be the case, the CSSD policy always leads to a reduction of the undesirable stationary mass. In this simulation, the SSD policy also always leads to therapeutic benefits. In general, the BOA, SSD, and CSSD control policies are all relatively conservative compared to the MFPT policy since the criteria they use are directly related to the network's long-run behavior.

We further consider performance comparison using 1000 randomly generated BNps with 7 genes and $p = 0.001$. For each network, without loss of generality, undesirable and desirable states are defined by $x_1 = 0$ and $x_1 = 1$, respectively, and the control gene is x_7. All four control policies are applied: MFPT with $\lambda = 0$; BOA; SSD; and CSSD. Table 3 summarizes the average stationary mass for the undesirable states before control (ORG) and after applying these four different policies.

Table 4 gives the percentages of random BNps with stationary mass shift $\Delta = \pi_U - \tilde{\pi}_U \geq 0$ for different control policies and different values of p_b.

10-gene random BNps

We consider performance comparison using 1000 randomly generated BNps with 10 genes and $p = 0.001$ in all experiments. For each network, without loss of generality, undesirable and desirable states are defined by $x_1 = 0$ and $x_1 = 1$, respectively, and the control gene is x_{10}. All four control policies are applied: MFPT with $\lambda = 0$; BOA; SSD; and CSSD. Table 5 summarizes the average stationary mass for the undesirable states before control (ORG) and after applying these four different policies.

Table 6 gives the percentages of random BNps with stationary mass shift $\Delta = \pi_U - \tilde{\pi}_U \geq 0$ for different control policies and different values of p_b.

Performance comparison for instantaneously random PBNs

7-gene randomly generated instantaneously random PBNs

We have also compared the performances using 1000 randomly generated instantaneously random 7–gene PBNs with 2 context BNps. We fix the perturbation probability at $p = 0.01$. The selection probabilities are $c_1 = c_2 = 0.5$. We again define states with $x_1 = 0$ as undesirable and states with $x_1 = 1$ as desirable, and again apply all four control policies with x_7 as the control gene. Table 7 summarizes the average stationary mass for the undesirable states before control (ORG) and after applying these four different control policies. The means and standard deviations of the stationary mass for undesirable states $\sum_{x_1=0} \pi_{\mathbf{x}}$ with $p_b = 0.5$ before and after control are shown in Fig. 2. The CSSD policy has the best performance and the SSD policy also achieves better performance compared with the MFPT and BOA policies, as in the simulations for BNps.

We also compute the percentages of random PBNs with stationary mass shift $\Delta = \pi_U - \tilde{\pi}_U \geq 0$ in Table 8 for different control policies and different p_b's. We see a similar trend as with the simulations for BNps.

We have further compared the performances using 1000 randomly generated instantaneously random 7–gene PBNs with 2 context BNps. We fix the perturbation probability at $p = 0.001$ this time. The selection probabilities are $c_1 = c_2 = 0.5$. We again define states with $x_1 = 0$ as undesirable and states with $x_1 = 1$ as desirable, and again apply all four control policies with x_7 as the control gene. Table 9 summarizes the average stationary mass for the undesirable states before control (ORG) and after applying these four different control policies.

We also compute the percentages of random PBNs with stationary mass shift $\Delta = \pi_U - \tilde{\pi}_U \geq 0$ in Table 10 for different control policies and different p_b's.

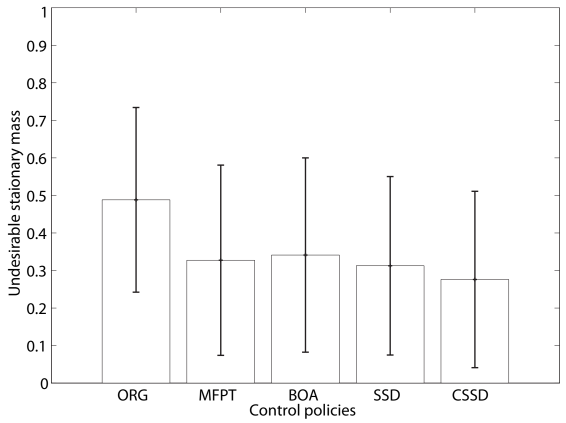

Figure 2: Performance comparison for 1000 randomly generated instantaneously random PBNs with $p_b = 0.5$ with respect to the means and standard deviations of the stationary masses for undesirable states for different control policies: ORG – original undesirable stationary mass; MFPT – undesirable stationary mass after applying the MFPT control policy; BOA – undesirable stationary mass after applying the BOA control policy; SSD – undesirable stationary mass after applying the steady-state distribution control policy; CSSD – undesirable stationary mass after applying the conservative SSD control policy.

10-gene randomly generated instantaneously random PBNs

We have compared the performances using 1000 randomly generated instantaneously random 10–gene PBNs with 2 context BNps. We fix the perturbation probability at $p = 0.001$. The selection probabilities are $c_1 = c_2 = 0.5$. We again define states with $x_1 = 0$ as undesirable and states with $x_1 = 1$ as desirable, and again apply all four control policies with x_{10} as the control gene. Table 11 summarizes the average stationary mass for the undesirable states before control (ORG) and after applying these four different control policies.

We also compute the percentages of random PBNs with stationary mass shift $\Delta = \pi_U - \tilde{\pi}_U \geq 0$ in Table 12 for different control policies and different p_b's.

References

[Brun *et al.*, 2005] Brun, M, Dougherty, ER, Shmulevich, I (2005) Steady-state probabilities for attractors in probabilistic Boolean networks, *Signal Processing*, **85**, 1993-2013.

[Faryabi *et al.*, 2009] Faryabi, B, Vahedi, G, Chamberland, J-F, Datta, A, Dougherty, ER (submitted) Intervention in context-sensitive probabilistic Boolean networks revisited.

[Faure *et al*., 2006] Faure, A, Naldi, A, Chaouiya, C, Theiffry, D (2006) Dynamical analysis of a generic Boolean model for the control of the mammalian cell cycle, *Bioinformatics*, **22**, 124-131.

[Pal *et al*., 2006] Pal, R, Datta, A, Dougherty, ER (2006) Optimal infinite horizon control for probabilistic Boolean networks, *IEEE Trans. on Sig. Proc.*, **54**, 2375-2387.

[Vahedi *et al*., 2008] Vahedi, G, Faryabi, B, Chamberland, J-F, Datta, A, Dougherty, ER (2008) Intervention in gene regulatory networks via a stationary mean-first-passage-time control policy, *IEEE Trans. on Biomed. Eng.*, **55**, 2319-2331.

Table 1: Average $\sum_{x_1=0} \pi_{\mathbf{x}}$ over 1000 randomly generated BNps with 7 genes ($p = 0.01$) before and after applying all of four control policies: ORG – original stationary mass for undesirable states before control; MFPT – mean-first-passage-time control policy; BOA – BOA control policy; SSD – steady-state distribution control policy; CSSD – conservative SSD control policy with x_7 as the control gene.

Control policies	p_b				
	0.1	0.3	0.5	0.7	0.9
ORG	0.8992	0.7128	0.5173	0.2965	0.0963
MFPT	0.8487	0.5428	0.3229	0.1619	0.0588
BOA	0.8542	0.5461	0.3216	0.1626	0.0583
SSD	0.8462	0.5211	0.3021	0.1491	0.0562
CSSD	0.8439	0.4884	0.2569	0.1348	0.0560

Table 2: Percentages of random BNps with $\Delta = \pi_U - \tilde{\pi}_U \geq 0$ within 1000 random BNps with 7 genes ($p = 0.01$) after applying four control policies with x_7 as the control gene.

Control policies	p_b				
	0.1	0.3	0.5	0.7	0.9
MFPT	96.8%	89.7%	88.5%	90.7%	97.2%
BOA	100.0%	99.3%	98.7%	99.3%	100.0%
SSD	100.0%	100.0%	100.0%	100.0%	100.0%
CSSD	100.0%	100.0%	100.0%	100.0%	100.0%

Table 3: Average $\sum_{x_1=0} \pi_{\mathbf{x}}$ over 1000 randomly generated BNps ($p = 0.001$) with 7 genes before and after applying all of four control policies: ORG – original stationary mass for undesirable states before control; MFPT – mean-first-passage-time control policy; BOA – BOA control policy; SSD – steady-state distribution control policy; CSSD – conservative SSD control policy with x_7 as the control gene.

Control policies	p_b 0.1	0.3	0.5	0.7	0.9
ORG	0.9090	0.7258	0.4885	0.2576	0.0908
MFPT	0.8627	0.5659	0.2927	0.1265	0.0424
BOA	0.8656	0.5676	0.2883	0.1280	0.0440
SSD	0.8590	0.5440	0.2745	0.1182	0.0410
CSSD	0.8576	0.5091	0.2185	0.1017	0.0410

Table 4: Percentages of random BNps with $\Delta = \pi_U - \tilde{\pi}_U \geq 0$ within 1000 random BNps with 7 genes ($p = 0.001$) after applying four control policies with x_7 as the control gene.

Control policies	p_b 0.1	0.3	0.5	0.7	0.9
MFPT	96.0%	87.0%	84.5%	92.5%	98.5%
BOA	100.0%	97.0%	95.0%	99.5%	100.0%
SSD	100.0%	100.0%	100.0%	100.0%	100.0%
CSSD	100.0%	100.0%	100.0%	100.0%	100.0%

Table 5: Average $\sum_{x_1=0} \pi_{\mathbf{x}}$ over 1000 randomly generated BNps with 10 genes ($p = 0.001$) before and after applying all of four control policies: ORG – original stationary mass for undesirable states before control; MFPT – mean-first-passage-time control policy; BOA – BOA control policy; SSD – steady-state distribution control policy; CSSD – conservative SSD control policy with x_{10} as the control gene.

Control policies	p_b				
	0.1	0.3	0.5	0.7	0.9
ORG	0.9088	0.7125	0.4837	0.2712	0.0924
MFPT	0.8757	0.5783	0.3002	0.1531	0.0590
BOA	0.8803	0.5901	0.3115	0.1553	0.0606
SSD	0.8729	0.5580	0.2917	0.1470	0.0589
CSSD	0.8714	0.5196	0.2240	0.1202	0.0581

Table 6: Percentages of random BNps with $\Delta = \pi_U - \tilde{\pi}_U \geq 0$ within 1000 random BNps with 10 genes ($p = 0.001$) after applying four control policies with x_{10} as the control gene.

Control policies	p_b				
	0.1	0.3	0.5	0.7	0.9
MFPT	97.4%	88.5%	88.0%	90.5%	98.3%
BOA	100.0%	97.4%	95.8%	98.3%	100.0%
SSD	100.0%	100.0%	100.0%	100.0%	100.0%
CSSD	100.0%	100.0%	100.0%	100.0%	100.0%

Table 7: Average $\sum_{x_1=0} \pi_{\mathbf{x}}$ over 1000 randomly generated PBNs with 7 genes ($p = 0.01$) before and after applying all of four control policies: ORG – original stationary mass for undesirable states before control; MFPT – mean-first-passage-time control policy; BOA – BOA control policy; SSD – steady-state distribution control policy; CSSD – conservative SSD control policy with x_7 as the control gene.

Control	p_b				
policies	0.1	0.3	0.5	0.7	0.9
ORG	0.9076	0.7071	0.4883	0.3014	0.0896
MFPT	0.8634	0.5686	0.3274	0.1654	0.0549
BOA	0.8676	0.5796	0.3412	0.1746	0.0564
SSD	0.8604	0.5512	0.3126	0.1547	0.0531
CSSD	0.8587	0.5297	0.2761	0.1406	0.0528

Table 8: Percentages of random PBNs with $\Delta = \pi_U - \tilde{\pi}_U \geq 0$ within 1000 random PBNs with 7 genes ($p = 0.01$) after applying four control policies with x_7 as the control gene.

Control	p_b				
policies	0.1	0.3	0.5	0.7	0.9
MFPT	96.5%	90.6%	92.7%	91.9%	96.1%
BOA	99.5%	96.1%	96.4%	96.5%	99.5%
SSD	100.0%	100.0%	100.0%	100.0%	100.0%
CSSD	100.0%	100.0%	100.0%	100.0%	100.0%

Table 9: Average $\sum_{x_1=0} \pi_{\mathbf{x}}$ over 1000 randomly generated PBNs with 7 genes ($p = 0.001$) before and after applying all of four control policies: ORG – original stationary mass for undesirable states before control; MFPT – mean-first-passage-time control policy; BOA – BOA control policy; SSD – steady-state distribution control policy; CSSD – conservative SSD control policy with x_7 as the control gene.

Control	p_b				
policies	0.1	0.3	0.5	0.7	0.9
ORG	0.9145	0.7220	0.5050	0.2858	0.0924
MFPT	0.8648	0.5790	0.3280	0.1519	0.0490
BOA	0.8643	0.5944	0.3373	0.1634	0.0524
SSD	0.8588	0.5585	0.3085	0.1407	0.0481
CSSD	0.8558	0.5345	0.2688	0.1236	0.0475

Table 10: Percentages of random PBNs with $\Delta = \pi_U - \tilde{\pi}_U \geq 0$ within 1000 random PBNs with 7 genes ($p = 0.001$) after applying four control policies with x_7 as the control gene.

Control	p_b				
policies	0.1	0.3	0.5	0.7	0.9
MFPT	94.6%	90.3%	90.7%	93.7%	96.7%
BOA	99.7%	94.9%	95.5%	96.3%	99.6%
SSD	100.0%	100.0%	100.0%	100.0%	100.0%
CSSD	100.0%	100.0%	100.0%	100.0%	100.0%

Table 11: Average $\sum_{x_1=0} \pi_{\mathbf{x}}$ over 1000 randomly generated PBNs with 10 genes ($p = 0.001$) before and after applying all of four control policies: ORG – original stationary mass for undesirable states before control; MFPT – mean-first-passage-time control policy; BOA – BOA control policy; SSD – steady-state distribution control policy; CSSD – conservative SSD control policy with x_{10} as the control gene.

Control policies	p_b				
	0.1	0.3	0.5	0.7	0.9
ORG	0.9133	0.7020	0.5067	0.2907	0.0888
MFPT	0.8777	0.5831	0.3505	0.1828	0.0516
BOA	0.8830	0.6004	0.3713	0.1963	0.0540
SSD	0.8752	0.5630	0.3389	0.1707	0.0504
CSSD	0.8734	0.5317	0.2924	0.1513	0.0499

Table 12: Percentages of random PBNs with $\Delta = \pi_U - \tilde{\pi}_U \geq 0$ within 1000 random PBNs with 10 genes ($p = 0.001$) after applying four control policies with x_{10} as the control gene.

Control policies	p_b				
	0.1	0.3	0.5	0.7	0.9
MFPT	95.5%	89.5%	91.1%	91.6%	96.4%
BOA	99.6%	93.2%	94.9%	95.7%	99.9%
SSD	100.0%	100.0%	100.0%	100.0%	100.0%
CSSD	100.0%	100.0%	100.0%	100.0%	100.0%
